# Supplementary material for: Assessment of Student Pharmacists’ Co-Curricular Professionalization Using an Impact Scale
Source: Pharmacy (Basel). 2024 Jul 25;12(4):117. doi: 10.3390/pharmacy12040117 (PMC11359949; doi:10.3390/pharmacy12040117)
Supplement: Supplementary file 1 [file pharmacy-12-00117-s001.zip › PhIT Co-Curricular Impact Scale 2023-24.pdf]

PhIT Portfolio Co-Curricular Impact Scale to Assess Participation in Co-Curricular Activities

"***After your participation in this activity, please answer the following questions, based on your impressions":***
1- Low Impact
2- Moderate Impact
3– Significant Impact
4– Not Applicable

1. This activity immersed me in an authentic learning experience. (CAPE 2.1, 2.3, 3.1)
2. This activity improved my self-confidence in providing patient care, advocacy, community service and/or leadership/service to the profession. (CAPE 3.3)
3. This activity improved my understanding and/or abilities to collaborate with other healthcare professions. (CAPE 3.4)
4. This activity allowed me to interact with patients and practice culturally sensitive care. This activity opened my eyes to provide care to patients who have cultural competency within pharmacy practice. (CAPE 3.5)
5. This activity allowed me to improve my communication skills with patients, colleagues, and/or other healthcare providers. (CAPE 3.6)
6. This activity was of high quality and will be referenced in my future professional interactions. (CAPE 4.1)
7. This activity made me feel more prepared for APPEs and becoming a pharmacist. (CAPE 4.1)
8. This activity increased my self-awareness such that I can more easily identify my strengths and weaknesses. (CAPE 4.1)
9. This activity coaxed me out of my "comfort zone" and promoted personal/professional growth. (CAPE 4.1)
10. This activity has opened my eyes to ideas/perspectives not previously recognized and stimulated intellectual curiosity. (CAPE 4.1)
11. This activity exposed me to potential career opportunities. (CAPE 4.1)
12. This activity will help me gain competency and facilitate life-long learning. (CAPE 4.1)
13. This activity allowed me to develop and refine my leadership abilities and skills. (CAPE 4.2)
14. This activity broadened my professional horizons and perspectives in areas of professionalism, altruism, accountability, and/or integrity. (CAPE 4.4)
15. This activity improved my academic/clinical knowledge related to pharmacy practice. (CAPE 1.1)
16. This activity allowed me to apply creativity, entrepreneurship, and/or an innovative mindset to address challenges and promote positive change. (CAPE 4.3)

Developed for 2021-22 PhIT Portfolio (Bach, Briceland, Veselov), Albany College of Pharmacy and Health Sciences; updated 2023-24

Please contact authors for permission to use Impact Scale (Kelly.Bach@acphs.edu; Laurie.Briceland@acphs.edu; Megan.Veselov@acphs.edu)
