# Supplementary material for: Assessment of Student Pharmacists’ Co-Curricular Professionalization Using an Impact Scale
Source: Pharmacy (Basel). 2024 Jul 25;12(4):117. doi: 10.3390/pharmacy12040117 (PMC11359949; doi:10.3390/pharmacy12040117)
Supplement: Supplementary file 1 [file pharmacy-12-00117-s001.zip › PhIT Portfolio2023-24.pdf]

Pharmacist- in- Training (PhIT) Portfolio 2023-24 (DUE DATE May 1, 2024)

Categories for Student Personal/Professional Development in Co-Curriculum	P1	P2	P3
*Students in the P1, P2 and P3 year must participate in a total **of 5 co-curricular events per academic year** from categories 1-5 below*<br>***NOTE**: Virtual/remote events will be acceptable in all categories*	**# of Entries within Portfolio**	**# of Entries within Portfolio**	**# of Entries within Portfolio**
1. **Professional Development/Education** (CAPE 4.1 [3,4,8]; 4.3.4; 4.4.5)<br>• Purpose: to set groundwork for selecting role-models, professional networking; to gain professional self-awareness/learn more about professional career options; to learn how innovation and entrepreneurship advances the profession through educational programming and career exploration events<br>• Examples: Attending educational events put on by professional organizations; attending Continuing Education or professional presentations at professional conferences (Local, state, national) or college symposia, education on digital health technologies<br>• *Career Exploration Event* may include: ACPHS Career Fair, Career Panels, Roundtable Events, guest speakers discussing career at professional organization meetings	1	1<br>Career Exploration Event	1
2. **Patient Care Service** (CAPE 2.3 [1,2]; 2.4.1; 3.2.5; 3.3.1;3.4.1;3.5.3)<br>• Purpose: directly interact with patients outside of classroom, work, or experiential setting<br>• Examples: Attendance at health screenings and fairs; "Brown Bags," blood pressure or immunization clinics, telehealth/telemedicine	Optional	Optional	1
3. **Legislative Advocacy** (CAPE 4.4 [3,5])<br>• Purpose: learn legislative process as it relates to pharmacy, how affected patients are in need of legislation, and how pharmacists can participate/make an impact<br>• Examples: Attend statewide Legislative Day activities or associated events on campus; educational sessions on legislative issues, etc.	1	1	1
4. **Leadership/Service to the Profession of Pharmacy** (CAPE 4.2[1,3,5];4.3[1,2,3,6) 4.4[1,2,5])<br>• Purpose: develop professional leadership skills; Learn how professional societies function; Give back to the profession; innovate/improve and/or implement professional events to advance profession of pharmacy<br>• Examples: Elected officer or other significant service to student professional organization; attendance or volunteer service at professional meetings; organization of meeting or event, team leader for an event or service; work to develop or improve a program or event; leadership seminar	Optional	1	Optional
5. **Healthcare-Related Community Service** (CAPE 2.3[1,2,4];3.2[3,5];3.3.1;3.5[2,3])<br>• Purpose: Civic responsibility to contribute/promote healthcare/wellness in community; be role model to others<br>• Examples: Healthcare-related fundraising events (e.g., Relay for Life, Making Strides walk, Pinky Swear Club events, etc.); medical mission (without academic credit), Panther Project; public outreach –educational events, creating "goody-bags" for patients, mission dinners	1	Optional	Optional
**Student's Choice from Categories 1-5**	**2 (to total of 5)**	**2 (to total of 5)**	**2 (to total of 5)**
Co-Curricular Professional Development Plan (Foundations of Pharmacy)	1		
Final PhIT Reflection (Spring P3)			1
**Resume/C.V. –** update each year – faculty advisor review/sign off, through CAMS	1	1	1

P1 Summary

- ☐ Complete Co-Curricular Professional Development Plan (submitted in Foundations of Pharmacy course)
- ☐ Participate in 5 co-curricular, PhIT approved events (1 Professional Development, 1 Legislative Advocacy, 1 Healthcare-Related Community Service, 2 of student's choice); Enter each event into Canvas
- ☐ CV/Resume; Review with Faculty Advisor during March meeting, enter into Canvas

P2 Summary

- ☐ Participate in 5 co-curricular, PhIT approved events (1 Career Fair/Panel, 1 Legislative Advocacy, 1 Leadership/Service to Profession of Pharmacy, 2 student's choice); Enter each event into Canvas
- ☐ Update CV/Resume; Review with Faculty Advisor during March meeting, enter into Canvas

P3 Summary

- ☐ Participate in 5 co-curricular, PhIT approved events (1 Professional Development, 1 Patient Care Service, 1 Legislative Advocacy, 2 student's choice); Enter each event into Canvas
- ☐ Update CV/Resume; Review with Faculty Advisor during March meeting, enter into Canvas
- ☐ Complete Final PhIT Reflection (Spring P3)

Additional Notes

- PhIT approved events are college sponsored co-curricular events, offered alongside the curriculum
- Non-college sponsored events must be approved through the "Non-college Sponsored Event Approval Form" in Canvas; one non-college sponsored event may be approved per student, per academic year
- Paid, work-related activities at a student's place of employment will not count toward PhIT requirements
- **Look for the "PhIT Approved" stamp on event posters and E-mails**

August 2023 M. Veselov; L. Briceland; J.Brewer portions adapted with permission from Western University; PhIT concept developed by Sarah Scarpace Peters
